# Establishment and application of a novel patient-derived KIAA1549:BRAF-driven pediatric pilocytic astrocytoma model for preclinical drug testing

**DOI:** 10.18632/oncotarget.14004

**Published:** 2016-12-17

**Authors:** Florian Selt, Juliane Hohloch, Thomas Hielscher, Felix Sahm, David Capper, Andrey Korshunov, Diren Usta, Sebastian Brabetz, Johannes Ridinger, Jonas Ecker, Ina Oehme, Jan Gronych, Viktoria Marquardt, David Pauck, Heidi Bächli, Charles D Stiles, Andreas von Deimling, Marc Remke, Martin U Schuhmann, Stefan M Pfister, Tilman Brummer, David T.W. Jones, Olaf Witt, Till Milde

**Affiliations:** ^1^ Clinical Cooperation Unit Pediatric Oncology (G340), German Cancer Research Center (DKFZ), and German Cancer Consortium (DKTK), Heidelberg, Germany; ^2^ Center for Individualized Pediatric Oncology (ZIPO) and Section of Pediatric Brain Tumors, Department of Pediatric Oncology, Hematology and Immunology, University Hospital Heidelberg, Heidelberg, Germany; ^3^ Division of Biostatistics (C060), German Cancer Research Center (DKFZ), Heidelberg, Germany; ^4^ Department of Neuropathology, University Hospital Heidelberg, Heidelberg, Germany; ^5^ Clinical Cooperation Unit Neuropathology (G380), German Cancer Research Center (DKFZ), and German Cancer Consortium (DKTK), Heidelberg, Germany; ^6^ Division of Pediatric Neurooncology (B062), German Cancer Research Center (DKFZ), Heidelberg, Germany, and German Cancer Consortium (DKTK), Heidelberg, Germany; ^7^ Division of Molecular Genetics (B060), German Cancer Research Center (DKFZ), and German Cancer Consortium (DKTK), Heidelberg, Germany; ^8^ AbbVie Deutschland GmbH & Co. KG, Medical Immunology, Wiesbaden, Germany (current affiliation); ^9^ Department of Pediatric Oncology, Hematology, and Clinical Immunology, Medical Faculty, University Hospital Düsseldorf, Germany, and Department of Pediatric Neuro-Oncogenomics, German Cancer Consortium (DKTK) and German Cancer Research Center (DKFZ), Heidelberg, Germany; ^10^ Department of Neurosurgery, University Hospital Heidelberg, Heidelberg, Germany; ^11^ Department of Cancer Biology, Dana-Farber Cancer Institute, Boston, MA, USA; ^12^ Department of Neurosurgery, University Hospital Tübingen, Tübingen, Germany; ^13^ Institute of Molecular Medicine and Cell Research, Albert-Ludwigs-University and University Medical Centre, Freiburg, Germany

**Keywords:** pediatric low grade glioma, pilocytic astrocytoma, KIAA1549:BRAF-fusion, oncogene-induced senescence (OIS), MAPK-inhibitors

## Abstract

Pilocytic astrocytoma (PA) is the most frequent pediatric brain tumor. Activation of the MAPK pathway is well established as the oncogenic driver of the disease. It is most frequently caused by KIAA1549:BRAF fusions, and leads to oncogene induced senescence (OIS). OIS is thought to be a major reason for growth arrest of PA cells *in vitro* and *in vivo*, preventing establishment of PA cultures. Hence, valid preclinical models are currently very limited, but preclinical testing of new compounds is urgently needed. We transduced the PA short-term culture DKFZ-BT66 derived from the PA of a 2-year old patient with a doxycycline-inducible system coding for Simian Vacuolating Virus 40 Large T Antigen (SV40-TAg). SV40-TAg inhibits TP53/CDKN1A and CDKN2A/RB1, two pathways critical for OIS induction and maintenance. DNA methylation array and KIAA1549:BRAF fusion analysis confirmed pilocytic astrocytoma identity of DKFZ-BT66 cells after establishment. Readouts were analyzed in proliferating as well as senescent states, including cell counts, viability, cell cycle analysis, expression of SV40-Tag, CDKN2A (p16), CDKN1A (p21), and TP53 (p53) protein, and gene-expression profiling. Selected MAPK inhibitors (MAPKi) including clinically available MEK inhibitors (MEKi) were tested *in vitro*. Expression of SV40-TAg enabled the cells to bypass OIS and to resume proliferation with a mean doubling time of 45h allowing for propagation and long-term culture. Withdrawal of doxycycline led to an immediate decrease of SV40-TAg expression, appearance of senescent morphology, upregulation of CDKI proteins and a subsequent G1 growth arrest in line with the re-induction of senescence. DKFZ-BT66 cells still underwent replicative senescence that was overcome by TERT expression. Testing of a set of MAPKi revealed differential responses in DKFZ-BT66. MEKi efficiently inhibited MAPK signaling at clinically achievable concentrations, while BRAF V600E- and RAF Type II inhibitors showed paradoxical activation. Taken together, we have established the first patient-derived long term expandable PA cell line expressing the KIAA1549:BRAF-fusion suitable for preclinical drug testing.

## INTRODUCTION

Pilocytic astrocytoma (PA) is a pediatric low-grade glioma (pLGG) and the most common pediatric brain tumor, accounting for ~18 % of all pediatric brain tumors [41]. It is a benign and slowly growing tumor corresponding to WHO grade I [31], which can arise anywhere in the CNS, but is most commonly localized in the cerebellum followed by the optic pathway/hypothalamic region [55]. Pilocytic astrocytomas almost never progress to higher grade astrocytomas, and dissemination occurs only in extremely rare cases [18]. Current standard of care therapy includes maximal safe resection where possible and chemotherapy and/or irradiation (e.g., SIOP LGG 2004 trial, NCT00276640). The ten-year overall survival (OS) rate of patients with PA exceeds 90% [18]. However, PAs can cause extensive morbidity due to local tumor expansion or therapy-related side effects (due to surgery, chemotherapy and irradiation), aggravated by recurrence or progressive disease (PD), which occurs in up to 80% of patients, depending on location and extent of initial resection [18]. Hence, PA comprises a chronic disease, and reduction of morbidity rather than increase of survival is the focus of current treatment strategies. The most commonly used chemotherapeutic agents, vincristine and carboplatin, are not effective in every patient, and PD is often observed during or after chemotherapy (ten-year event-free survival (EFS) rate of 32%) [18]. Therefore, the development of new therapies is urgently needed in order to specifically target the disease and improve the clinical course of patients suffering from PA.

Recent years of research have uncovered the underlying genetic alterations of PA. Initial studies described the prototypic KIAA1549-BRAF fusion [10, 24, 42], and recent large scale genomic profiling approaches identified alternative, yet less frequent events [23, 68]. The most frequent alteration found in PA is the activating fusion of KIAA1549 with the BRAF kinase domain (>60%), followed by typically mutually exclusive BRAF^V600E^ point mutation, FGFR1 mutations, NTRK-family fusions, NF1 mutations, or KRAS mutations [23, 68]. All these alterations lead to an activation of the mitogen-activated protein kinase (MAPK) signaling pathway, which is detected in nearly 100% of cases, identifying PA as a single pathway disease [23, 68].

All of the identified alterations are targetable, either directly or by inhibiting activity downstream of the alteration [43]. Specific targeted drugs are clinically available, since they have already been approved for the treatment of different malignancies or are in preclinical and phase I/II clinical trials (e.g. BRAF-inhibitors) [47].

The first compound targeting BRAF that entered a phase II clinical trial for pediatric LGG patients was sorafenib [25]. Sorafenib is a multi-kinase inhibitor targeting BRAF, VEGFR, PDGFR and c-kit. The trial failed due to unexpected tumor growth under sorafenib treatment, which was explained by paradoxical MAPK pathway activation through facilitated BRAF dimer formation in the presence of the drug [51]. This experience highlighted the urgent need for detailed preclinical testing of every new compound before entering a clinical trial, and for more detailed knowledge of the mechanism of action of each individual drug.

Comprehensive preclinical drug tests for PAs are currently hampered by the lack of suitable preclinical models. Although PA is the most frequent pediatric brain tumor, only a few preclinical models have been published. In 2011, Gronych et al. reported on a mouse model where RCAS-mediated gene delivery of a truncated version of BRAF^V600E^-kinase reliably generated PA-like tumors *in vivo* [19]. In the same year Raabe et al. published a PA model, where transduction of BRAF^V600E^ into human neural stem cells caused transformation and subsequent growth arrest [44]. These models replicate BRAF^V600E^ positive PAs, however they are difficult to expand and therefore only of limited use for medium- to high-throughput preclinical testing. The only patient-derived long-term pediatric LGG cell line that is available to date is BT-40 [3]. This model was established from a juvenile pleomorphic xanthoastrocytoma patient and is characterized by a BRAF^V600E^ mutation and homozygous CDKN2A/B deletion, thereby molecularly resembling a WHO grade II-III glioma rather than a PA. To date, there are no reported patient-derived PA cell lines, and consequently no model with endogenous expression of the prototypical KIAA1549:BRAF fusion on a human genetic background [39]. Due to this lack of true KIAA1549:BRAF fusion-positive PA models all preclinical data on the fusion was generated using models where it was artificially overexpressed, e.g. in fibroblasts. [24, 51]. However, these models do not recapitulate the expression levels of the fusion in PAs, and do not exhibit the cellular background of PAs.

Our own efforts to generate PA models by orthotopical transplantation of primary PA tumor material into mice in order to generate patient-derived xenografts (PDX) or by cultivating primary PA cells *in vitro* under neural stem cell conditions failed in 36/36 cases. In comparison, the take rate of orthotopically transplanted high-grade gliomas in mice was ~30% in our hands (unpublished observation). A possible reason for the failure of PA model generation was identified by the detection of oncogene-induced senescence (OIS) in the vast majority of PA tumor samples, primary short-term cultures and *in vitro* models [22, 44]. OIS is a form of premature senescence found in benign RAS and RAF driven tumors [34, 49], among others. It is accompanied by accumulation of p53 and p16 (CDKN2A) [49] leading to permanent cell cycle arrest. OIS is thought to be a tumor-suppressive mechanism preventing tumors from further malignant transformation in the absence of additional cooperating mutations and serves as an explanation for the benign nature of PA with almost no tendency to malignant transformation. Since OIS is clearly detectable upon culture of primary PA cells [22], we hypothesized that inducible interference with the OIS program can reversibly bypass growth arrest in primary PA cells, enabling the establishment of a long-term expandable cell line.

In order to reversibly suppress OIS, a lentiviral doxycycline-inducible expression system coding for Simian Vacuolating Virus 40 large T antigen (SV40-TAg) was generated. The viral protein SV40-TAg inhibits two of the major pathways involved in the induction and maintenance of OIS, TP53/CDKN1A and CDKN2A/RB1 [2, 9]. Using this tool we generated a novel patient-derived PA model, DKFZ-BT66, with endogenous expression of the KIAA1549:BRAF fusion and maintenance of typical PA characteristics, suitable for long-term expansion and preclinical drug screening.

## RESULTS

### Doxycycline-dependent expression of SV40-TAg in DKFZ-BT66 leads to long-term proliferation

**Figure 1:**
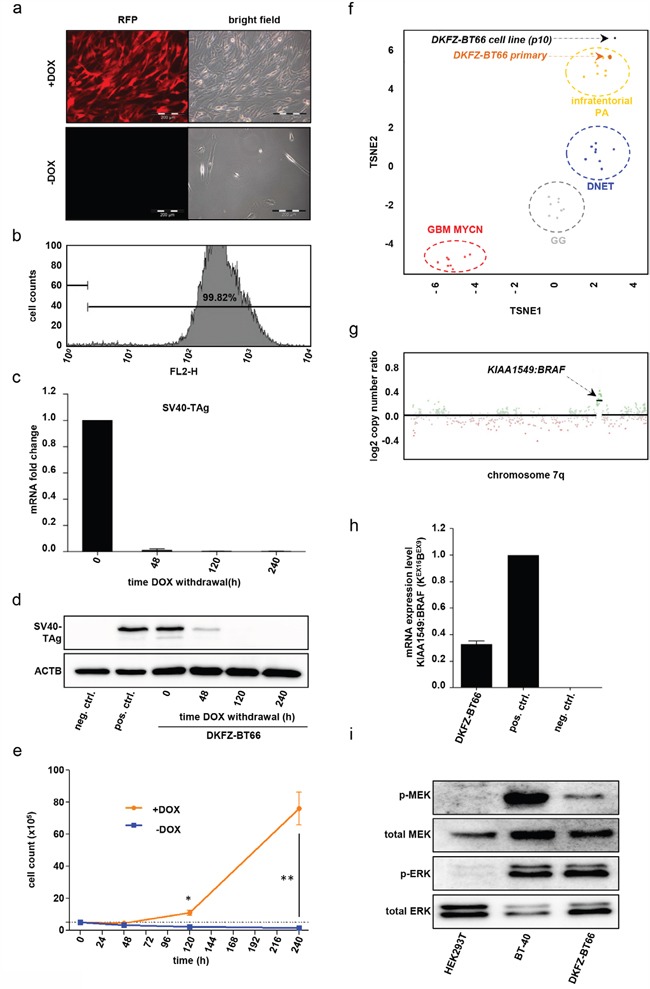
**a**. Light and fluorescence microscopy: DKFZ-BT66 cells cultured in the presence of 1μg/ml doxycycline for 10 days (upper row) show marked expression of RFP indicating activity of the inducible promotor and enhanced proliferation as opposed to cells cultured in the absence of doxycycline, which do not express RFP. **b**. Flow cytometric detection of red fluorescence indicating RFP positivity in DKFZ-BT66 cells cultured in the presence of 1 μg/ml doxycycline. A single population emerged after puromycin selection exhibiting high fluorescence levels, as demonstrated by 99,8% of the cells displaying RFP (FL2-H) positivity (representative plot shown). **c**. Fold change of SV40-TAg mRNA level after the indicated time points of doxycycline withdrawal compared to cells cultured in the presence of 1μg/ml doxycycline (time point 0). Depicted are mean +/− SD **d**. Change in SV40-TAg protein level in DKFZ-BT66 cells upon doxycycline withdrawal. HEK293T cells, constitutively expressing SV40-TAg, served as positive control (pos. ctrl.); BT-40 glioma cells, not expressing SV40-TAg, were used as negative control (neg. ctrl.). **e**. Growth kinetics of cells cultured in the presence (yellow line) or absence (blue line) of doxycycline, respectively. Dashed line indicates the number of cells seeded at hour 0. Significant differences between the groups are indicated * p<0.05 and ** p<0.01. Depicted are mean +/− SD **f**. TSNE analysis (t-Distributed Stochastic Neighbor Embedding) of whole genome DNA methylation data. DKFZ-BT66 cell line (passage 10, black dot) exhibited a close similarity in DNA-methylation to its primary tumor (orange dot) and the group of infratentorial pilocytic astrocytomas but differed from other pediatric low- and high grade tumors. DNET: Dysembryoplastic neuroepithelial tumor, WHO grade I; GG: Ganglioglioma, WHO grade I; GBM MYCN: MYCN-amplified glioblastoma, WHO grade IV. **g**. Copy number plot of chromosome 7q calculated from DNA-methylation array data confirming the presence of a chromosomal gain typical for the tandem duplication that leads to a KIAA1549:BRAF-fusion. DNA was extracted from passage 10 cells. **h**. Detection of KIAA1549:BRAF(K^EX16^B^EX9^)-fusion gene mRNA by RT-qPCR using fusion break point specific primers at passages 10, 12 and 15. Mean +/− SD of expression at the three passages is depicted. An astrocyte cell line (TÜ-DKFZ-002) transduced with a retroviral construct coding for the full length form of KIAA1549:BRAF(K^EX16^B^EX9^)-fusion served as positive control (pos. ctrl.) and reference for expression level. Neuroblastoma cell line BE-(2)C served as a fusion negative control (neg. ctrl.) **i**. MAPK downstream activation status of DKFZ-BT66 in the presence of 1μg/ml doxycycline on pMEK (Ser217/ Ser221) and pERK (Thr202/ Tyr204) level compared to the BRAF^V600E^-mutated PXA cell line BT40 (positive control) and the BRAF-wild type cell line HEK293T (negative control).

In order to generate an expandable and experimentally functional *in vitro* model of PA, we performed lentiviral transduction of DKFZ-BT66 cells at passage 2 with a tetracycline-inducible vector (pFRIPZ TAg) co-expressing red fluorescent protein (RFP) and SV40-TAg. SV40-TAg targets the OIS mediators RB1 and TP53, thereby inhibiting induction of OIS [2, 9]. DKFZ-BT66 cells were cultured in medium supplemented with doxycycline, allowing for doxycycline-induced co-expression of SV40-TAg and RFP. Doxycycline-induced minimal-CMV promoter activity was detectable *in vitro* by fluorescence microscopy of RFP expression (Figure 1a). In contrast, RFP expression was not detectable by immunofluorescence microscopy after 12 days of culture without doxycycline, indicative of reduced promotor activity (Figure 1a). Flow cytometry documented a highly enriched RFP-expressing population after puromycin selection of transduced DKFZ-BT66 cells under doxycycline (Figure 1b). SV40-TAg expression upon addition of doxycycline was time- and concentration dependent as measured on mRNA and protein levels. Withdrawal of doxycycline from the culture medium led to a considerable decrease of SV40-TAg mRNA level after 48h (Figure 1c). Accordingly, SV40-TAg protein levels were strongly decreased by 48h and undetectable by 120h after doxycycline withdrawal (Figure 1d). A comparable reduction of SV40-TAg mRNA and protein level was seen in cells cultured at decreased concentration of doxycycline for 5 days (Supplementary Figure 1a-1b). While addition of 1 μg/ml doxycycline resulted in SV40-TAg protein levels comparable to positive control HEK293T cells (constitutively expressing SV40-TAg), almost no SV40-TAg protein was detectable at concentrations as low as 0.1 μg/ml doxycycline.

DKFZ-BT66 cells demonstrated steady proliferation with a calculated mean doubling time of 43.8h (SD 3.8; n=3, passage 11 - 13) in the presence of doxycycline and hence SV40-TAg expression (Figure 1e). Of note, loss of SV40-TAg expression upon withdrawal of doxycycline resulted in complete proliferation arrest of DKFZ-BT66 without significant changes in total cell counts over 240h, after an initial drop in cell numbers. Comparison of cells with or without SV40-TAg expression revealed statistically significant differences in cell counts from 120h onwards (Figure 1e).

Taken together, our system allows for precise experimental control of doxycyclin-induced SV40-TAg expression in DKFZ-BT66 cells, effectively blocking growth arrest and inducing cell proliferation.

### DKFZ-BT66 reflects the typical molecular and cellular biology of pilocytic astrocytoma

In order to confirm the PA origin of isolated DKFZ-BT66 cells, we investigated DNA-methylation patterns, sequencing of the KIAA1549:BRAF fusion, fusion transcript expression, and MAPK pathway activation. Genome-wide methylation analysis using methylation arrays has previously been shown to be a robust tool to pinpoint identity and molecular subsets of a wide range of brain tumors including PAs [30], glioblastomas [57], medulloblastomas [21] and tumors designated as “PNET” [56]. t-SNE analysis of genome-wide methylation data confirmed that the primary DKFZ-BT66 tumor sample belongs to the methylation group of infratentorial PA. It furthermore revealed that the DKFZ-BT66 cell line at passage 10 still exhibited a closer similarity to the methylation group of infratentorial PAs compared to other methylation clusters of pediatric low- as well as high-grade tumors. This demonstrates that the methylation pattern of the cell of origin inherent to the primary tumor remained conserved following *in vitro* culture, transfection as well as antibiotic selection procedures (Figure 1f).

DKFZ-BT66 cells were derived from a primary PA tumor exhibiting the typical and specific KIAA1549:BRAF-fusion (K^EX16^B^EX9^). Indeed, analysis of the copy number plot derived from the DNA methylation data of the DKFZ-BT66 cells revealed the presence of the prototypical ~2Mb duplication on chromosome 7q34 leading to KIAA1549:BRAF fusion (Figure 1g). The increased magnitude of gain seen in the cells compared with the primary DKFZ-BT66 tumor sample indicated a high tumor cell purity of the cultured DKFZ-BT66 cells (Supplementary Figure 1c). The presence of the KIAA1549:BRAF-fusion (K^EX16^B^EX9^) was additionally confirmed by gene-panel sequencing with detected breakpoints within BRAF intron 8 (position 140488009 on chromosome 7) and KIAA1549 intron 16 (position 138538359 on chromosome 7) (not shown). The K^EX16^B^EX9^ transcript was detected continuously over several passages (p10, p12 and p15) in DKFZ-BT66 cells by RT-qPCR using fusion-spanning primers specific for K^EX16^B^EX9^(Figure 1h). In line with the presence and previously reported consequence of the KIAA1549:BRAF-fusion, MAPK downstream activation was detected by Western Blot in DKFZ-BT66 cells under SV40-TAg expressing proliferating conditions (Figure 1i). DKFZ-BT66 cells showed higher levels of phosphorylated MEK (pMEK; Ser217/ Ser221) and ERK (pERK; Thr202/ Tyr204) compared to the BRAF wild type cell line HEK293T constitutively expressing SV40-TAg, and an ERK activation comparable to the BRAF^V600E^-expressing cell line BT-40 (Figure 1i). Although we could clearly demonstrated the PA origin of DKFZ-BT66 cells, some additional copy number aberrations not present in the primary tumor and rather atypical for PA (e.g. partial loss of chromosome 17q) were detected in the cell line (Supplementary Figure 1d). These changes remained stable over several passages, however, and no further appreciable changes occurred at higher passages.

In summary, DKFZ-BT66 displayed a methylation profile similar to primary infratentorial PAs as well as to the primary tumor of origin, expressed and maintained the prototypical KIAA1549:BRAF fusion, and showed the expected MAPK activity. Thus, we conclude that DKFZ-BT66 cells are indeed a true PA model.

### Growth arrest of DKFZ-BT66 cells in the absence of SV40-TAg is accompanied by a senescence phenotype

**Figure 2:**
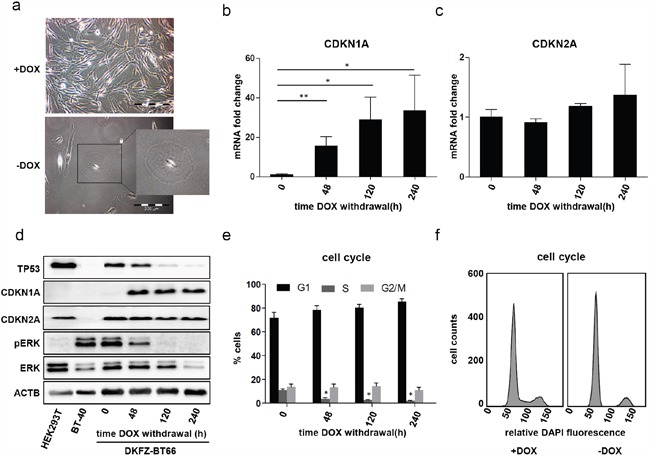
**a**. Light microscopic comparison of passage 10 DKFZ-BT66 cells grown in the presence of doxycycline (+DOX; 1 μg/ml) and cells cultivated in the absence of doxycycline (-DOX) for 21 days. Cells grown in the absence of doxycycline show enlarged, round and flattened cell bodies as compared to a small spindle like phenotype observed in cells proliferating in the presence of doxycycline. **b, c**. Fold changes of CDKN1A (p21) and CDKN2A (p16) mRNA in DKFZ-BT66 cells after doxycycline withdrawal normalized to the level in cells cultured in the presence of 1 μg/ml doxycycline (0 hours). n=3, significant differences are indicated as: ** p<0.01 and * p<0.05. Depicted are mean +/− SD **d**. Western blot analysis of total cell lysates. DKFZ-BT66 cells were seeded in full medium supplemented with 1μg/ml doxycycline and medium was replaced by doxycycline-free medium 24 hours later. Cells were lysed after the indicated time in the absence of doxycycline. BT-40 and HEK293T were cultured in normal growth medium without doxycycline before lysis **e**. Changes in cell cycle distribution after the indicated culture time in the absence of doxycycline. An increase in G1-phase and a significant reduction (*p<0.05) in S-phase cells was detected as compared to control (0 hours) (n=3). Depicted are mean +/− SD f. Representative cell cycle histograms of DKFZ-BT66 cells cultured in the presence (+DOX) or absence (-DOX for ten days) of doxycycline for ten days, respectively.

Proliferation of DKFZ-BT66 cells was strongly SV40-TAg dependent, with loss of SV40-TAg expression resulting in growth arrest. As expression of SV40-TAg was used in order to bypass OIS, this phenotype was not unexpected and in line with the re-induction of OIS. It was accompanied by several senescence-associated phenotypic changes of DKFZ-BT66 cells, comparable to primary PA cultures in OIS: withdrawal of doxycycline led to a distinct change in cell morphology with rounded, enlarged and flattened cells (Figure 2a), a phenotype typical for senescent cells [49]. A statistically significant induction of the cyclin dependent kinase inhibitor (CDKI) CDKN1A (p21) expression was detectable on mRNA and protein level 48h after doxycycline withdrawal, but not for the CDKI CDKN2A (p16) (Figure 2b-2d). The expected accumulation of TP53 in the presence of SV40-TAg [40] steadily decreased in a time-dependent manner after loss of SV40-TAg expression upon doxycycline withdrawal (Figure 2d). The time-dependent decrease of pERK with a complete loss of pERK after 120h indicated abrogation of MAPK-activation in the absence of SV40-TAg (Figure 2d). Cell cycle analysis revealed a cell cycle arrest with an increase in G0/G1-phase and a decrease of S-phase upon loss of SV40-TAg expression 48 hours after doxycycline withdrawal and at later time points (Figure 2e, 2f).

**Figure 3:**
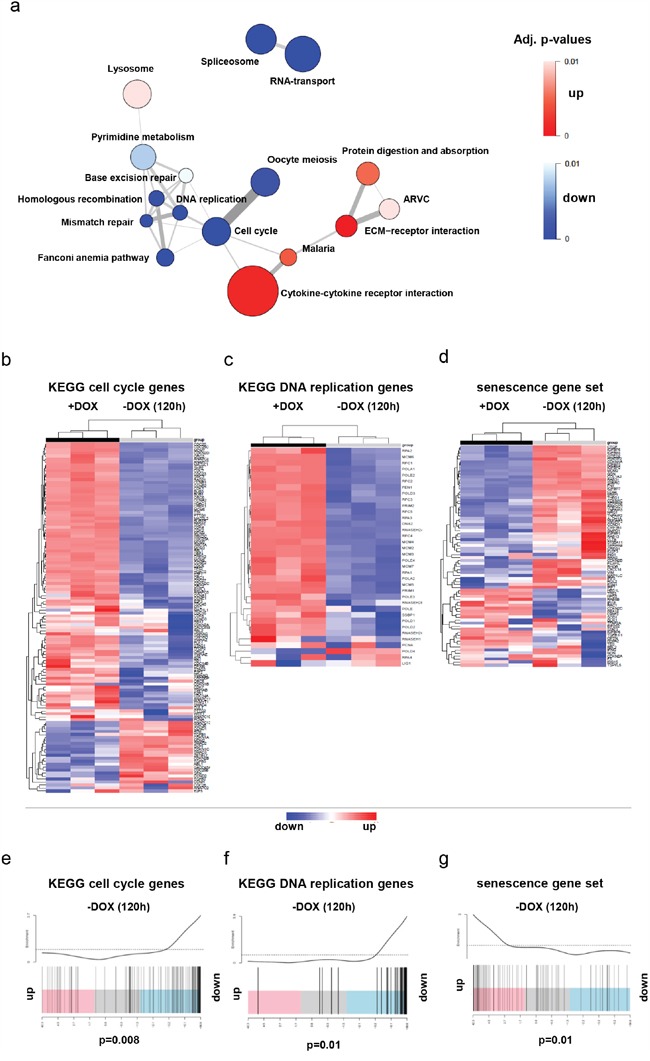
Gene expression profiling (GEP) data of DKFZ-BT66 was obtained in the presence (+DOX, n=3) or absence (-DOX, n=3) of doxycycline for five days. **a**. Pathway enrichment map derived from GEP data showing the most regulated (up and down) KEGG pathways in senescent (-DOX) DKFZ-BT66 cells (p< 0.01). The size of the circles is proportional to the number of genes in the respective pathway. The width of the edges (lines connecting two circles) is proportional to the number of genes overlapping between the two pathways. **b, c, d**. The heatmaps show the regulation of genes summarized as cell cycle genes (b) and DNA replication genes (c) by the KEGG database and a senescence gene set (d) published by Fridman et al [20]. Unsupervised clustering separated the 3 +DOX and the 3 -DOX samples in each case and indicated differential expression of the analyzed genes in the two settings. Most of the genes of the cell cycle (b) and DNA replication (c) gene sets were downregulated in the absence of doxycycline. In contrast, the genes of the senescence gene set were predominantly upregulated in the absence of doxycycline. **e, f, g**. Gene set enrichment analysis performed on the same gene sets confirmed significant downregulation of cell cycle (e, p= 0.008) and DNA replication genes (f, p= 0.01) in the absence of doxycycline and upregulation of senescence genes (g, p= 0.01).

In order to characterize the transcriptomic changes upon induction of cell cycle arrest after loss of SV40-TAg expression, we compared gene expression profiles of DKFZ-BT66 cells expressing SV40-TAg (with doxycycline) and without SV40-TAg expression (120h after doxycycline withdrawal). Pathway enrichment analysis of genes regulated upon SV40-TAg loss revealed a significant downregulation of pathways involved in cell cycle, DNA replication and RNA processing, and an upregulation of pathways known to be associated with senescence such as cytokine-cytokine receptor interaction [29] (Figure 3a Table 1). Unsupervised clustering of the KEGG gene sets “cell cycle” (hsa04110) and “DNA replication“ (hsa03030), as well as a senescence gene set published by Fridman et al. [15] revealed a very clear separation of the samples into two different groups according to induction or absence of SV40-TAg expression (Figure 3b-3d). The majority of the genes in the gene sets “cell cycle” and “DNA replication” were downregulated in the group without SV40-TAg expression, whereas the majority of the genes in the senescence gene set was upregulated. Accordingly, gene set enrichment analyses (GSEA) performed on the same three gene sets (Figure 3e-3g) confirmed a statistically significant enrichment of downregulated genes of the gene sets “cell cycle”, “DNA replication” and of upregulated senescence genes in cells upon SV40-TAg loss.

**Table 1:** The top 10 most differentially regulated KEGG pathways in DKFZ-BT66 in proliferating vs. arrested mode

KEGG Pathway	No. of genes	Direction of regulation	P-Value	FDR
DNA replication	36	Down	1,35E-13	4,02961E-11
Cell cycle	118	Down	1,448E-09	1,62706E-07
ECM-receptor interaction	61	Up	1,63798E-09	1,62706E-07
Spliceosome	116	Down	2,87395E-08	2,14109E-06
RNA transport	136	Down	3,81383E-08	2,27304E-06
Fanconi anemia pathway	46	Down	4,50544E-07	2,2377E-05
Mismatch repair	22	Down	1,97183E-06	8,39436E-05
Cytokine-cytokine receptor interaction	151	Up	2,02138E-05	0,000752964
Homologous recombination	28	Down	4,74498E-05	0,001441792
Oocyte meiosis	99	Down	4,83823E-05	0,001441792

Direction of regulation in the senescence mode is shown.

A generally accepted feature of cells undergoing OIS is their positive staining for SA- β-galactosidase [6, 11]. Indeed, DKFZ-BT66 cells showed marked activity of SA- β-galactosidase in the absence of doxycycline, confirming their senescent state. Circumvention of growth arrest by expression of SV40-Tag, however, was not sufficient to abrogate SA- β-galactosidase staining of DKFZ-BT66 cells, suggesting that not all features of senescence are reverted in our model system (Supplementary Figure 1e).

Taken together, the observed growth arrest of DKFZ-BT66 in the absence of SV40-TAg was accompanied by distinct morphological as well as cell biological changes indicative of induction of a senescent phenotype comparable to OIS.

### Growth arrest upon loss of SV40-TAg expression is not due to replicative senescence

Growth arrest accompanied by senescent features can be due to both oncogene-induced as well as replicative senescence. An ultimate growth arrest of DKFZ-BT66 cells was observed after 19 (+/− 1) passages (n=3 replicates) independent of the presence of doxycycline. This was accompanied by morphologic changes reminiscent of senescence with flattened, enlarged and irregularly-formed cell bodies (Supplementary Figure 2a-2b). Since the promoter driving SV40-TAg expression was still active, as indicated by RFP expression (Supplementary Figure 2b), we presumed that senescence at this point could not be reverted by SV40-TAg and was therefore different from OIS. We hypothesized that the observed growth arrest was therefore replicative (instead of oncogene-induced) senescence, the former of which is reversible by human telomere reverse transcriptase (TERT) expression. We transduced DKFZ-BT66 (passage 18) cells with a retroviral expression system coding for TERT (pBabe_hygro_hTERT), leading to constitutive TERT expression (Supplementary Figure 2d). Of note, untransduced DKFZ-BT66 cells showed no endogenous TERT expression on mRNA level (Supplementary Figure 2d). TERT expression allowed DKFZ-BT66 PA cell line to proliferate beyond passage 20 (up until passage 32 at the time of manuscript submission) and prevented senescent changes, preserving a cell morphology similar to early passage cells (supplementary Figure 2c), suggestive of a reversion of replicative senescence. Loss of SV40-TAg by withdrawal of doxycycline still led to a subsequent and robust growth arrest independent of TERT expression (Supplementary Figure 2e). Of note, TERT cells grew slightly faster in the presence of SV40-TAg (doubling time: 41.7 +/− 6.7 h) as compared to DKFZ-BT66 cells without TERT overexpression (Figure 1e). We conclude that TERT expression inhibited replicative senescence but not senescence induced by loss of SV40-TAg expression.

In summary, these data demonstrate that DKFZ-BT66 cells carry a dominant senescence program that differs from replicative senescence and is in line with OIS driven by the KIAA:BRAF1549 fusion.

### A DKFZ-BT66-derived PA OIS gene signature identifies PA patients with excellent progression free survival

**Figure 4:**
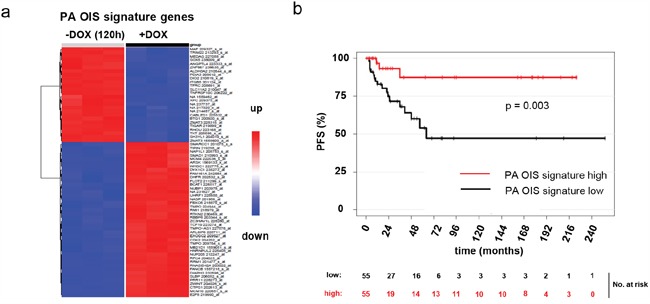
A PA OIS signature was derived from gene expression data of DKFZ-BT66 in proliferation (+DOX) and senescence mode (-DOX). **a**. Heatmap of the 68 PA OIS signature probesets. **b**. Kaplan-Meier curves depicting the progression free survival in patients of the ICGC PedBrain PA cohort with either high or low expression of the PA OIS signature genes. Patients with high OIS score show a significantly increased progression free survival as compared to patients with a low OIS score (p=0.003).

In order to identify, which patients are reflected by our model in either state (proliferating or in OIS), we generated a gene signature for OIS in PA based on DKFZ-BT66 cells in either proliferation or OIS. A penalized logistic regression model was fitted resulting in a PA OIS signature of 68 probesets (Figure 4a; Supplementary Table 3). This gene signature was applied to a dataset of 112 clinically and molecularly annotated primary PA samples (pilocytic astrocytoma ICGC PedBrain cohort [23]) (Table 2). The signature was able to discriminate between two subsets of patients within this cohort with significantly different PFS (Figure 4b). Patients with low expression of the PA OIS signature had a significantly decreased PFS rate, as opposed to patients with a high expression (p=0.003). There was no significant correlation of the OIS signature with other parameters annotated in the primary PA dataset.

**Table 2:** Annotations of the Pilocytic Astrocytoma ICGC PedBrain cohort

		n	% of total
age	median: 8 years (0.8-26)		
	pediatric (<18 years)	100	89,3
	adult (18 years and older)	12	10,7
gender	male	49	43,8
	female	63	56,3
location	infratentorial	84	75,0
	spinal	1	0,9
	brain stem	12	10,7
	cerebellum	68	60,7
	supratentorial	27	24,1
	optic pathway	6	5,4
	3rd ventricle	4	3,6
	4th ventricle	4	3,6
	diencephalon	8	7,1
	cerebral hemisphere	7	6,3
MAPK alteration	BRAF-fusion	92	82,1
	BRAF-mutation	6	5,4
	FGFR1-mutation	5	4,5
	NF1	2	1,8
	KRAS-mutation	2	1,8
	NTRK2-fusion	1	0,9
	NA	4	3,6
resection status	total	61	54,5
	subtotal	34	30,4
	NA	17	15,2

We conclude that DKFZ-BT66 reflects patients with a higher risk of progression in the proliferating state, and patients with a low risk of progression in the senescent state. Therefore, proliferating DKFZ-BT66 need to be applied for drug testing, since they reflect the patients at risk of progression and in need of further therapy.

### DKFZ-BT66 identifies MEK inhibitors as promising candidates for the treatment of proliferating pilocytic astrocytoma

**Figure 5:**
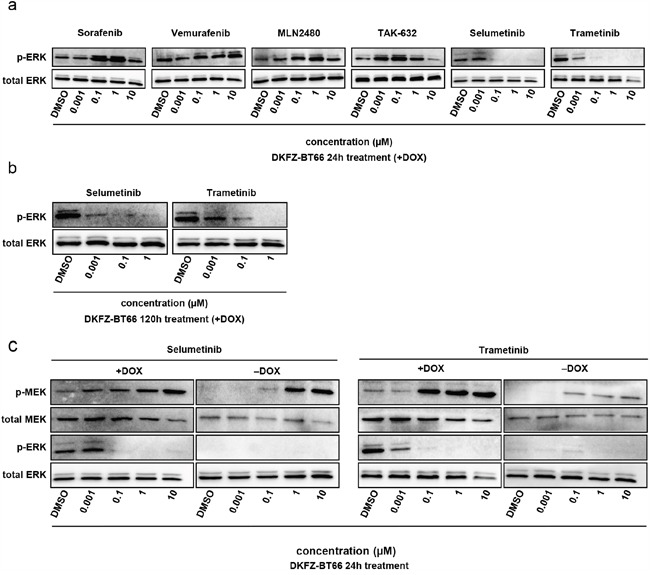
**a**. Western blot analysis of total cell lysates from DKFZ-BT66 cells treated for 24 hours with the indicated inhibitors before lysis. For each condition 2 x10^6^ cells were seeded 24 hours before treatment in 10 cm culture dishes (Corning) in ABM supplemented with doxycycline **b**. Western blot analysis of total cell lysates from cells treated for 120 hours with selumetinib or trametinib before lysis. For each condition 2 x10^6^ cells were seeded 24 hours before treatment in 10 cm culture dishes in ABM supplemented with doxycycline. 48-72 hours after the beginning of treatment medium was exchanged by fresh medium containing the indicated concentrations of inhibitor and 1μg/ml doxycycline in order to keep inhibitor- and doxycycline levels s. Western blot analysis of total cell lysates from cells treated for 24 hours with selumetinib or trametinib. For experiments in proliferation (+DOX) 2×10^6^ cells were seeded 24 hours before treatment in 10 cm culture dishes in ABM supplemented with 1μg/ml doxycycline and then treated for 24h with the inhibitors before lysis. For experiments in senescence mode (-DOX) 4×10^6^ cells were seeded in 10 cm cell culture dishes and cultivated in the absence of doxycycline for five days followed by treatment with selumetinib or trametinib for 24 hours before lysis.

Inhibitors of the MAPK pathway represent a promising class of drugs currently under investigation for the treatment of tumors with MAPK activation (reviewed in Samatar et al. [47]). We tested a set of clinically available and investigational MAPK inhibitors (MAPKi) - BRAF inhibitors (BRAFi), inhibitors of dimeric RAF (RAFi), and MEK inhibitors (MEKi) - for their ability to inhibit MAPK-signaling, metabolic activity and proliferation in DKFZ-BT66 cells. Two approved BRAFi, sorafenib (a multi-kinase inhibitor also targeting RAF kinases) and vemurafenib (a BRAF V600E-specific inhibitor), caused paradoxical activation of the MAPK-signaling cascade as indicated by an increase in pERK level after 24h of treatment (Figure 5a). This has been suggested earlier in other BRAF-fusion-driven models [25, 51]. MLN2480 and TAK-632, two potent class II RAFi [47], also caused paradoxical activation of the pathway in our model (Figure 5a). Conversely, ERK activation was fully abrogated by two clinically available MEKi, selumetinib and trametinib, at concentrations as low as 0.1 μM at 24h (Figure 5a), as indicated by loss of pERK signal in the Western blot. The loss of pERK persisted upon long-term treatment of 120h, without signs of paradoxical activation (Figure 5b). Both MEKi increased the level of pMEK after 24h in a concentration dependent manner, in both SV40-TAg expressing and non-expressing settings, indicative of feedback activation (Figure 5c). Of note, measurement of MEKi effects on pERK by western blots in the senescent cells was precluded due to the previously demonstrated loss of pERK after induction of senescence (Figure 5c).

**Figure 6:**
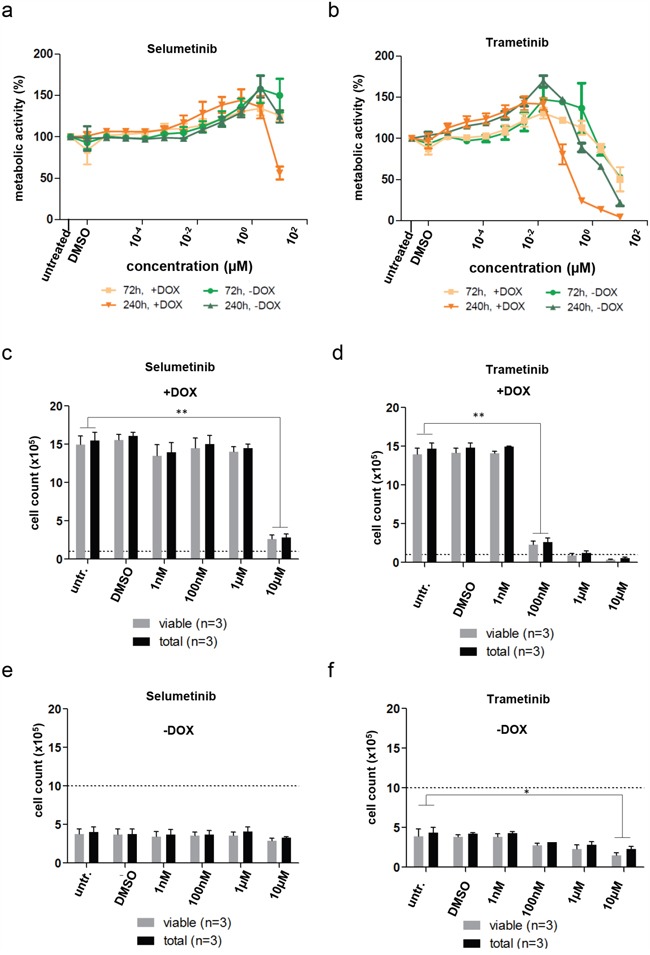
**a, b**. Assessment of metabolic activity by CellTiter-Glo assay. Mean of three independent replicates +/− SD is depicted. a) Cells were treated with selumetinib for 72 and 240 hours, respectively, either in proliferation (+DOX, 1 μg/ml doxycycline) or senescence mode (-DOX). b) Cells were treated with trametinib for 72 and 240 hours, respectively, either in proliferation (+DOX, 1 μg/ml doxycycline) or senescence mode (-DOX). **c-f**. Influence of inhibitor treatment on proliferation of DKFZ-BT66 in proliferation (+DOX) or senescence mode (-DOX). Cells were treated for 240 hours with the indicated concentrations and then counted by automated trypan blue staining. Data is mean +/− SD of three biological replicates. Significant differences are indicated as: ** p<0.01 and * p<0.05. Dashed lines indicate the number of cells seeded per well of a 6-well plate (Corning) 24 hours before treatment was started.

By design of our model, all effects mediated by TP53/CDKN1a or CDKN2a/RB1 are blocked in the proliferating state, since both pathways are blocked by SV40-TAg. Accordingly changes in metabolic activity as well as cell numbers upon treatment with both MEKis studied did not correlate well with the observed effects on pERK reduction. Both MEKi caused an unexpected concentration-dependent increase of metabolic activity in DKFZ-BT66 cells treated at lower doses for 72 or 240 hours (Figure 6a-6b), which was not caused by increased cell counts (Figure 6c-6d). Reduction of metabolic activity and cell counts after long-term treatment (240 hours) in the proliferating state was more pronounced in cells treated with trametinib than in cells treated with selumetinib (Figure 6a-6d). The effects on proliferation of DKFZ-BT66 cells treated in senescent mode (loss of SV40-TAg expression without doxycycline) were weaker or not detectable (Figure 6e-6f).

In summary, we demonstrated differential responses to inhibitors of different points in the MAPK signaling cascade, with BRAFi and RAFi activating and MEKi inhibiting the pathway.

## DISCUSSION

Pilocytic astrocytoma is the most common pediatric brain tumor but no patient-derived model of this entity suitable for long-term use has been established up to now. Several of our own efforts to generate PA models either *in vivo* or *in vitro* had also previously failed. We hypothesized that OIS, which was found to be present in PA patient samples and short-term cultures [22], is the main hurdle for successful model development. In line with this hypothesis, inhibition of senescence mediators RB1 and TP53 by expression of SV40-TAg in short-term cultured PA cells enabled us to circumvent senescence and to establish a proliferating PA cell line. The resulting cell line, DKFZ-BT66, preserved a typical molecular and cell biological phenotype characterizing it as true PA. DKFZ-BT66 exhibits several advantages compared to already existing non-patient-derived model systems: I) DKFZ-BT66 displays the true genetic background of a PA as opposed to the available models that are based on non-PA cells such as neural stem cells [44], fibroblasts [24, 51] or murine neural stem cells [19]. II) DKFZ-BT66 is the first cell line endogenously expressing the prototypical KIAA1549:BRAF fusion. It therefore recapitulates the PA fusion expression level much closer than the existing KIAA1549:BRAF-models which are mainly based on fusion-overexpressing fibroblasts. III) The expandability of existing models can be limited due to senescence (as seen in primary PA cell cultures), potentially limiting their use for medium- and large-scale experiments. Circumvention of senescence in DKFZ-BT66 abrogates this limitation and allows for expansion of the cell line.

The inducibility of the expression system used in our model allowed for reversion of SV40-TAg expression by withdrawal of doxycycline. Loss of SV40-TAg expression was accompanied by several senescence features, including enlarged cell morphology, upregulation of CDKN1a, and G0/G_1_ cell cycle arrest [49]. Discrimination between OIS and replicative senescence can be challenging, since both of them share a subset of mediators (e.g. TP53, CDKN1A, RB1, CDKN2A and others) and result in a similar phenotype [28]. However, our functional evidence indicates that OIS is present in DKFZ-BT66 cells after loss of SV40-TAg expression. Replicative senescence occurs when telomeres are shortened, ultimately resulting in crisis when induction of senescence is inhibited [32]. Stabilization of telomeres is therefore a critical step in the development of malignant tumors [32]. Many tumor cells gain immortality by expression of telomerase [50] or use alternative lengthening of telomeres (ALT) to maintain their telomeres [36] to avoid replicative senescence and crisis. Expression of TERT was absent in DKFZ-BT66 and the cells entered growth arrest after about 20 passages in culture despite SV40-TAg expression, indicative for crisis being present in these late passage cells. The fact that no spontaneous immortalized clone evolved from DKFZ-BT66 cells in crisis (more than 3 months of observation) indicates that DKFZ-BT66 cells did not reactivate TERT or initiate ALT. This observation is in line with a previously published study by Tabori et al., which demonstrated the absence of TERT activity or ALT and therefore continuous shortening of telomeres over time in pilocytic astrocytoma tumor samples [59]. Artificial overexpression of telomerase in our model allowed the cells to overcome ultimate growth arrest, to proliferate beyond their replicative lifespan and to maintain initial cell shape, functionally proving that the absence of telomerase activity and/or ALT in DKFZ-BT66 was responsible for late passage growth arrest. Proliferation of TERT-overexpressing DKFZ-BT66 cells remained strongly dependent on SV40-TAg expression. TERT expression cannot bypass OIS, since the trigger of OIS is independent from telomeres [64], but is capable of a durable reversion of replicative senescence and crisis since telomere shortening is their trigger [4, 61]. Growth arrest observed in TERT-overexpressing DKFZ-BT66 after SV40-TAg withdrawal was therefore strongly suggestive for OIS being present in DKFZ-BT66. As the additional expression of hTERT was only intended for experimental purposes, future screenings should not be performed on DKFZ-BT66 transduced with hTERT without testing for differences in biological readouts beforehand.

Of note, CDKN2A levels did not change after doxycycline withdrawal. This was not unexpected, since SV40-Large T inhibits the CDKN2A/RB1 pathway at the level of RB1, which is downstream of CDKN2A and therefore does not influence the expression level of CDKN2A. On the other hand, CDKN1A is affected since SV40-TAg inhibits p53 upstream of CDKN1A. Therefore CDKN1A goes up again after p53 is released from inhibition and can affect it´s downstream targets.

We observed that MAPK activation was downregulated after induction of senescence. Senescence was maintained in the absence of MAPK signaling, indicating that oncogenic signaling via ERK is only needed for induction of senescence, but not for its maintenance. One possible mechanistic explanation for downregulation of MAPK activity in senescence might be the upregulation of ERK specific phosphatases (e.g. DUSP family members) and other regulatory proteins via e.g. TP53 as has been described before in other OIS systems [7]. Further investigations need to be performed to explain this observation in the DKFZ-BT66 model. However, MAPK silencing in arrested cells compared to proliferating cells might indicate that the use of MAPKi is more likely to be successful in proliferating cells since the target for MAPKi is expressed to a lesser extent in senescent cells. MAPKi therefore might influence PA only at the time of progression or the proliferating subset of cells within a tumor, which is rather low according to immunohistochemical Ki67 detection of PA samples (typically <5%) [12, 17]. This could explain the delay of several months that is seen until first clinical responses under treatment with MEKis is detected (unpublished observation).

The observation of OIS being present and reversible in DKFZ-BT66 makes it well suitable for studies on OIS in PA, and led us to develop a PA OIS gene signature. Application of this signature on gene expression data of a cohort of PA patients allowed us to define two groups of patients, statistically significantly different in their PFS. Patients with low PA OIS score had a significantly decreased PFS rate, as opposed to patients with a high PA OIS score. We concluded that DKFZ-BT66 in proliferating or senescent state models PA in either progressing mode or OIS, respectively. Low or absent OIS activity correlates with an activated MAPK pathway in DKFZ-BT66. Patients with low PA OIS score therefore could represent a subset in which MAPK activation and susceptibility to MEK inhibition is higher, highlighting the potential for these drugs in the treatment of progressing patients and giving a possible biomarker for predicting the subset of patients most likely to show a response. The potential use of the PA OIS score as an independent prognostic marker has to be investigated prospectively within clinical trials.

As a single pathway disease characterized by activation of the MAPK pathway, inhibition of MAPK is a promising novel approach to PA treatment. Careful preclinical testing of new inhibitors before application in clinical trials is strongly needed. This has been highlighted by a study investigating the RAF inhibitor sorafenib, which reported unexpected tumor growth in pediatric PA patients in a phase II trial [25]. Paradoxical activation of the MAPK signaling pathway was identified as the molecular correlate of the observed increase in tumor mass. DKFZ-BT66 was able to detect paradoxical activation after treatment with sorafenib, recapitulating the results of the phase II trial [25], and the same was observed for vemurafenib [51]. Since KIAA1549:BRAF fusions signal as homodimers [51], we tested MLN2480 and TAK-632, two potent inhibitors of dimeric RAF [47], for their activity in DKFZ-BT66. However, both MLN2480 and TAK-632 caused unexpected paradoxical activation of pERK in our model system, similar to sorafenib and vemurafenib. Other groups have reported MAPK inhibition upon RAFi treatment in RAS and BRAF mutated as well as BRAF fusion backgrounds. MLN2480 inhibited MAPK pathway signaling in preclinical BRAF and RAS mutant melanoma and colorectal carcinoma models [8]. In addition, MLN2480 has recently been shown to suppress pERK and cell proliferation in short term cultures of primary pilocytic astrocytoma cells that were confirmed as positive for the KIAA1549:BRAF fusion gene [58]. TAK-632 was initially described to have cellular activity against mutated BRAF or mutated NRAS cancer cell lines and antitumor efficacy *in vivo* in BRAF and NRAS mutated xenograft models [38]. TAK-632 was shown to induce minimal paradoxical activation and potent antiproliferative effects in preclinical NRAS- and BRAF-mutated melanoma models [37]. Finally, TAK-632 showed MAPK inhibitory effects with loss of pERK in a SND1:BRAF fusion pancreatic cancer model [5]. Conversely, in our model setting treatment with RAFi led to MAPK activation. We therefore conclude that the lack of MAPK inhibition upon RAFi treatment could be a class-specific aspect of our model system. One possible explanation of this finding is the described paradoxical activation of downstream ERK signaling upon RAF inhibition in conditions of elevated RAS-GTP [26], which needs to be investigated in future studies.

In contrast, two MEKi, selumetinib and trametinib, caused robust and durable downregulation of MAPK activity in DKFZ-BT66, in line with reported clinical responses of LGGs to MEK inhibitors in phase I studies (unpublished data, and [1, 65]). These findings support the investigation of MEKi in current phase I/II clinical trials and their potential as candidates for translation into upcoming phase III clinical trials. Both trametinib and selumetinib inhibited MAPK signaling efficiently at clinically achievable concentrations, as measured by reduction of pERK levels. However, this did not translate into simultaneous reduction of cell numbers or metabolic activity. The reduction of cell number and metabolic activity after treatment with trametinib but not selumetinib is inconsistent with the effective abrogation of MAPK signaling under treatment with either compound at the same concentrations. Thus the observed effect could be of unspecific toxic nature. We believe the most likely reason for the lack of observable inhibition of cell growth attributable to MEKi to be model-inherent: while SV40-TAg inhibits OIS, by its very function it abrogates TP53 function and thereby inhibits all TP53-dependent downstream effects. This is enforced by SV40-TAg acting as a powerful mitogen, which has been shown to induce cell growth in growth factor free medium [54]. As SV40-TAg acts downstream of the MAPK-signaling cascade, it could have such a strong autonomous growth promoting effect that inhibition of MEK upstream of SV40-TAg cannot be fully translated into the desired effect on viability and metabolic activity. Another explanation of the unchanged or increased metabolic activity and unchanged cell numbers at MEKi concentrations clearly inhibiting MAPK activity is that activation of parallel signaling pathways including the PI3K-mTOR pathway leads to resistance to MEKi [66]. PI3K activation has been shown to overcome BRAF V600E-induced senescence in melanoma [63], and PI3K signaling was found to be enhanced in BRAFi-resistant melanomas [62]. Other mechanisms could include the reconfiguration of chromatin leading to sustained up-regulation of mechanisms activating RAS signaling through PI3K, as has been shown in IDH mutant gliomas [14], with subsequent stimulation of metabolism. Lastly, the lack of specific effects on cell counts and metabolic activity could be a function of time, and this could very well be expected. In patients clinical response delay is seen even under chemotherapy, and a response to MEKi in early trials has been noted to be measurable after months of treatment. This is very well conceivable given the slow-growing nature of PAs, and furthermore emphasizes the preclinical validity of this model. However, preclinical drug screens using this model will have to take the limitation into account, that signaling effects such as changes in pMEK and pERK are readily detectable, but effects on cell proliferation or cell death are limited. Further efforts to generate new patient-derived and true PA models with different genetic backgrounds are urgently warranted, hopefully overcoming the limitation of our model.

In summary, the DKFZ-BT66 model is an *in vitro* PA-model well suited for testing of compounds inhibiting MAPK signaling based on BRAF fusion-driven activation. It will be also very useful to understand the biochemistry of BRAF fusion proteins that significantly differ in their regulatory requirements from BRAF point mutants such as V600E [27]. It is capable of preclinical identification of MAPKi leading to either robust inhibition of MAPK pathway activity or paradoxical activation on a faithful genetic and epigenetic PA background. Finally, the model is fully suitable for OIS studies in PA.

## MATERIALS AND METHODS

### Ethics statement

Investigation has been conducted in accordance with the ethical standards and according to the Declaration of Helsinki and according to national and international guidelines and has been approved by the authors' institutional review board.

### Patient sample

The pilocytic astrocytoma cell line DKFZ-BT66 was derived from a primary tumor sample of a pilocytic astrocytoma patient obtained during therapeutic intervention by one of the co-authors (MUS). Informed consent for sample collection and use was obtained, and the study was approved by the institutional review board of the University of Heidelberg (S-304/2014). The male patient was almost 2 years of age (22 months) when he was diagnosed with a partially cystic left cerebellar tumor causing a strong dislocation of the brain stem and a compression of the mesencephalon. The diagnosis of pilocytic astrocytoma was confirmed by a local neuropathologist and by a central German histopathology review board. The KIAA1549:BRAF fusion (K^EX16^B^EX9^) was detected by the presence of a specific fusion transcript. After gross total resection of the tumor, no further therapeutic intervention was necessary. The patient is being monitored on a regular basis, and up to now (4 years and 8 months after resection) no relapse of the disease has occurred.

### Viral vectors and transduction

The lentiviral vector pFRIPZ was generated from the pTRIPZ plasmid (Open Biosystems), which contains a doxycycline regulated promotor (minimal CMV) controlling a turboRFP cDNA followed by a shRNAmir cassette. To generate pFRIPZ, the *NotI* site in pTRIPZ was eliminated and the mir30 based shRNAmir cassette was replaced by the encephalomyocarditis virus internal ribosomal entry site (EMCV IRES) followed by an *EcoRI* and *NotI* cutting site for subcloning. SV40-TAg DNA was amplified from retroviral pQCXIH/TAg vector [45] using primers with *NotI* overhangs and sub-cloned into pJET cloning vector (pJET1.2/blunt, Thermo Fisher Scientific, Cat. No. K1231) by blunt end ligation using manufacturers’ instructions. SV40-Tag-containing pJET plasmids were digested with NotI and cut SV40-TAg inserts were separated on a 1% agarose gel. pFRIPZ vector was digested with *NotI* and ligated with SV40-TAg DNA. Clones with correctly orientated SV40-TAg inserts were identified by restriction digest and positive clones were sequenced via an external sequencing service (GATC Biotech, Germany) and used for further plasmid preparations. pBABE-hygro-hTERT plasmid was a gift from Bob Weinberg (Addgene plasmid # 1773). The full open reading frame (LKB) of the KIAA1549:BRAF-fusion (K^EX16^B^EX9^) was described before [24] and subcloned into retroviral pBABE-hygro plasmid (pBABE-hygro-LKB).

Lentiviral packaging of pFRIPZ TAg vector was performed using Thermo Scientific Trans-Lentiviral Packaging system (Cat. No. TLP5912). To this end, 5 × 10^6^ HEK293T cells grown in a 10 cm culture dish for 24h were co-transfected with pFRIPZ TAg plasmid DNA and Trans-Lentiviral Packaging Mix using calcium phosphate transfection. Retroviral packaging was performed using Platinum-GP Retrovirus Expression System, Pantropic from Cell Biolabs (VPK-302). Therefore, 5 × 10^6^ Platinum-GP cells grown in a 10 cm culture dish for 24h were co-transfected with pBABE-hygro-hTERT or pBABE-hygro-LKB plasmid DNA and pCMV-VSV-G plasmid DNA using calcium phosphate transfection.

Medium was changed 16-20 hours after transfection, and viral supernatant was collected 24h after the medium change. Polybrene (Santa Cruz) was added to a final concentration of 8μg/ml and viral supernatant was used for immediate target cell transduction or stored in aliquots at -80°C. Target cells were seeded at a density of 5 × 10^5^ cells in 10 cm culture dishes 24 hours prior to infection followed by incubation with three ml viral supernatant. After six hours, a top-up with seven ml target cell medium was added. Antibiotic selection of transduced clones with either puromycin (pFRIPZ TAg plasmid, 1μg/ml, Cayman Chemicals, catalogue no. 13884) or hygromycin-B (pBABE-hygro-hTERT and pBABE-hygro-LKB plasmids, 250μg/ml, Santa Cruz, catalogue no. sc-29067) was started 24 hours after infection and performed for 10 days.

### Cell culture

DKFZ-BT66 cell line was cultivated as a monolayer in ABM basal medium (LONZA; CC-3187, US) supplemented with AGM SingleQuot Kit Supplements & Growth Factors (LONZA; CC-4123, US). For expansion, 1μg/ml doxycycline (Santa Cruz; sc-337691) was added to the medium prior to use and medium was replaced three times a week to obtain stable doxycycline levels. DKFZ-BT66 cells were passaged every 7-10d into new cell culture dishes after short enzymatic digestion with 0.05% trypsin-EDTA (Gibco, UK). For experiments in the absence of SV40-TAg, DKFZ-BT66 cells were seeded in complete growth medium without doxycycline. BT-40 cell line [3] was a kind gift of Dr. P. Houghton, University of Texas Health Science Center at San Antonio, and was grown in RPMI1640 with L-glutamine (LONZA; BE12-702F, Belgium) and 10% fetal bovine serum (Sigma Aldrich, Germany). DKFZ-BT66 cells and BT-40 cells have been proven to be free of contamination by mycoplasma or viral contamination using the Multiplex cell Contamination Test (McCT)[48]. Confirmation of identity by Multiplex Cell line authentication test (MCA)[48] was not possible since these cell lines are not present in the MCA database. HEK293T cells (GE Healthcare, Dharmacon catalogue no. HCL4517) were propagated in DMEM medium with L-glutamine (LONZA; BE12-604F, Belgium) supplemented with 10% fetal bovine serum (Sigma Aldrich, Germany) and had identity confirmed and proven to be free of contamination by mycoplasma or viral contamination using the Multiplex Cell Contamination and Authentication Test (MCA and McCT)[48]. Platinum GP cells (Cell Biolabs, catalogue no. RV-103) were propagated in DMEM medium with L-glutamine (LONZA; BE12-604F, Belgium) supplemented with 10% fetal bovine serum (Sigma Aldrich, Germany) and 10μg/ml blasticidin (Gibco, UK). TÜ-DKFZ pBABE-LKB, a cell line derived from non-neoplastic brain artificially overexpressing the KIAA1549:BRAF fusion, was cultivated in ABM basal medium (LONZA; CC-3187, US) supplemented with AGM SingleQuot Kit Supplements & Growth Factors (LONZA; CC-4123, US). All cell lines were grown at 37°C in a humidified atmosphere with 5% CO_2_. Cell counts were performed by automated trypan blue staining using Vi-CELL XR automated cell counter (Beckmann Coulter, Germany). Doubling time of cultured cells was calculated using publicly available software (http://www.doubling-time.com/compute_more.php). All cell lines in culture were checked regularly every month for contamination with mycoplasma using Venor®GeM Mycoplasma PCR Detection Kit (Minerva Biolabs, 11-1250, Germany).

### MAPK inhibitors

All inhibitors were purchased from Selleckchem. A panel of RAF inhibitors including sorafenib (catalogue no. S1040), vemurafenib (catalogue no. S1267), MLN2480 (catalogue no. S7121) and TAK-632 (catalogue no. S7291) and two MEK inhibitors, selumetinib (catalogue no. S1008) and trametinib (catalogue no. S2673), were investigated. All compounds were ordered as 10mM stock solution dissolved in DMSO and stored at -80°C in 50μl aliquots until used. Inhibitors and solvent control (0.1% DMSO) were diluted in cell culture medium (with or without doxycycline depending on the context) and added to the cell culture at the indicated concentrations for the indicated time.

### Metabolic activity

Measurement of metabolic activity in DKFZ-BT66 cells was performed in 96-well flat bottom black opaque wall plates (Greiner) after inhibitor treatment for 72 or 240 hours. 24 hours before treatment 25.000 cells/well were seeded for experiments with 72 hours duration and 5.000 cells/well were seeded for experiments with 240 hours duration in experiments in the presence of 1μg/ml doxycycline. For experiments in the absence of doxycycline 100.000 cells/well were seeded 24 hours prior to treatment for both, the short and the long-term treatment time. Cells were incubated with the different MAPK inhibitors in several dilutions, ranging from 10 μM to 0.005 nM. All treatments were performed in triplicate. Each plate contained an untreated and a DMSO control. Medium was exchanged three times per week and replaced in order to keep inhibitor levels and doxycycline levels stable. After the incubation time metabolic activity was determined using CellTiter-Glo assay (Promega, catalogue no. G7571) following manufacturer´s instructions. Luminescence was detected by FLUOstar OPTIMA automated plate reader (BMG Labtech). Dose-response curves and half maximal inhibitory concentrations (IC_50_-values) were calculated using GraphPad Prism software (v5.01) for windows.

### Western blot

Western blot analysis was performed as described previously [13]. In short, protein concentrations of cell lysates were determined using the Thermo Scientific Pierce (Waltham, MA, USA) BCA Protein Assay Kit according to manufacturer's instructions. The antibodies used are summarized in supplementary Table 1. Luminescence signals were detected using Amersham ECL Prime Western Blotting Detection System (GE Healthcare) on PVDF membrane with Chemi- Smart 5000 Technology (Vilber Lourmat).

### RNA-isolation, cDNA synthesis and quantitative reverse transcription real-time PCR (qPCR)

RNA extraction from cell culture and cDNA synthesis was performed as reported previously [35]. Quantitative real-time PCR was conducted using an ABI 7500 Real Time PCR cycler (Applied Biosystems) using ABI 7500 Software v2.3 (Applied Biosystems) with Platinum SYBR Green qPCR SuperMix-UDG (Invitrogen). The quantitative real-time PCR conditions were as described previously [35]. Quantitative real-time PCR Primers were purchased from Qiagen and Invitrogen (see supplementary Table 2). The ΔΔCt method was used to assess relative quantification. *ACTB* was used as control gene.

### Microscopy

Bright field pictures were captured as described before [13]. Fluorescence images of RFP expressing cells were captured using an Olympus CX41 microscope in combination with an Olympus U-RFL-T reflected fluorescence system and CellB 2.3 software. Exposure time was 25ms for bright field and 500ms for fluorescence images.

### Cell cycle analysis

1.5 x10^6^ cells were washed with PBS and fixed by dropwise addition of 70% ice cold ethanol while vortexing. Following 30 minutes incubation on ice cells were washed once and re-suspended in staining solution containing 0.1% Triton-X-100 and 1μg/ml 4',6-Diamidino-2-Phenylindole (DAPI; Applichem). After 30 minutes incubation at room temperature, cells were analyzed on a BD FACS Canto II flowcytometer using a 405 nm excitation laser and a Pacific-Blue emission filter. Doublets were discriminated using a SSC-W/SSC-H plot and cell cycle was plotted as counts/Pacific-Blue histogram. Evaluation of data was performed using FlowJo-V10 software.

### Senescence-associated β-galactosidase staining

Senescence-associated (SA) β-galactosidase staining was performed using a senescence β-galactosidase staining kit (Cell Signaling Technology, catalogue no. 9860) following manufacturer´s instructions.

### DNA-methylation array and t-SNE analysis

The Illumina Infinium HumanMethylation450 Beadchip (450k) array was used for molecular subgrouping and copy number profiling as described before [21]. t-Distributed Stochastic Neighbor Embedding (t-SNE) analysis was performed as described before [56].

### Gene panel sequencing

A customized gene panel covering 130 brain tumor-related genes was used as described [46]. In brief, a target enrichment method based on the Agilent SureSelect Enrichment technology (Santa Clara, CA, USA) was applied. The generated libraries (enriched regions of interest) cover the exonic regions of the respective genes, as well as intronic regions involved in gene fusions including KIAA1549:BRAF breakpoints.

### Gene expression profiling, pathway analysis, GSEA and development of a PA OIS gene signature

Gene expression profiling was performed as described before [23]. Hierarchical clustering was performed using Euclidean distance and complete linkage after gene-wise standardization. Differentially expressed probesets were identified using the empirical Bayes approach [53] based on moderated t-statistics as implemented in the Bioconductor package limma [52]. KEGG pathways (http://rest.kegg.jp) and a previously published senescence pathway [15] were used for pathway analysis. In case multiple probesets mapped to the same gene, the probeset with the largest effect based on limma analysis was used. The camera test [67] was used to competitively test pathways for regulation between conditions. All p-values were adjusted for multiple testing using Benjamini-Hochberg correction in order to control the false discovery rate. An enrichment map [33] was used to visualize results from pathway tests. A penalized logistic regression model was fitted on DKFZ-BT66 samples to find a PA OIS signature using the glmnet algorithm [16]. Due to the small sample size, an elastic net penalty was used to allow for a moderately sized signature. A small elastic net mixing parameter (α=0.99) was added to the L1 penalty parameter. The L1 penalty parameter was determined based on leave-one-out cross-validation. The OIS score was predicted on PA patient samples and scaled. Prognostic impact of OIS score on progression-free survival was assessed using Kaplan-Meier estimates and log-rank test. OIS score was dichotomized at median cut-off. P-values below 0.05 were considered statistically significant. All analyses were carried out using R 3.2 [60].

### Statistics

*In vitro* experiments were performed in a minimum of three biological replicates. All data is presented as mean ± standard deviation (SD). Differences between two groups were compared using an unpaired t-test with Welch´s correction. P-values <0.05 were considered significant. Dose-response curves and half-maximal inhibiting concentrations (IC_50_) were calculated using GraphPad Prism version 5.01 (GraphPad Software, La Jolla, CA, USA) for Windows. Graphs were generated using GraphPad Prism version 5.01 and Microsoft Powerpoint 2010 for Windows.

## SUPPLEMENTARY MATERIALS FIGURES AND TABLES





## Abbreviations

ALT: alternative lengthening of telomeres
BRAFi: BRAF inhibitor
CDKI: cyclin dependent kinase inhibitor
DAPI: 4',6-Diamidino-2-Phenylindole
EFS: event-free survival
GSEA: gene set enrichment analysis
IC50: half-maximal inhibiting concentration
MEKi: MEK inhibitors
MAPK: Mitogen-activated protein kinase
MAPKi: MAPK inhibitor(s)
OIS: oncogene induced senescence
PA: pilocytic astrocytoma
PD: progressive disease
PDX: patient-derived xenografts
pLGG: pediatric low-grade glioma
RAFi: RAF inhibitor(s)
RFP: red fluorescent protein
SA: Senescence-associated
SD: standard deviation
SV40-TAg: Simian Vacuolating Virus 40 large T antigen
TERT: telomere reverse transcriptase
