# Supplementary material for: Establishment and application of a novel patient-derived KIAA1549:BRAF-driven pediatric pilocytic astrocytoma model for preclinical drug testing
Source: Oncotarget. 2016 Dec 17;8(7):11460–79. doi: 10.18632/oncotarget.14004 (PMC5355278; doi:10.18632/oncotarget.14004)
Supplement: Supplementary file 1 [file oncotarget-08-11460-s001.pdf]

Establishment and application of a novel patient-derived KIAA1549:BRAF-driven pediatric pilocytic astrocytoma model for preclinical drug testing

SUPPLEMENTARY FIGURES AND TABLES

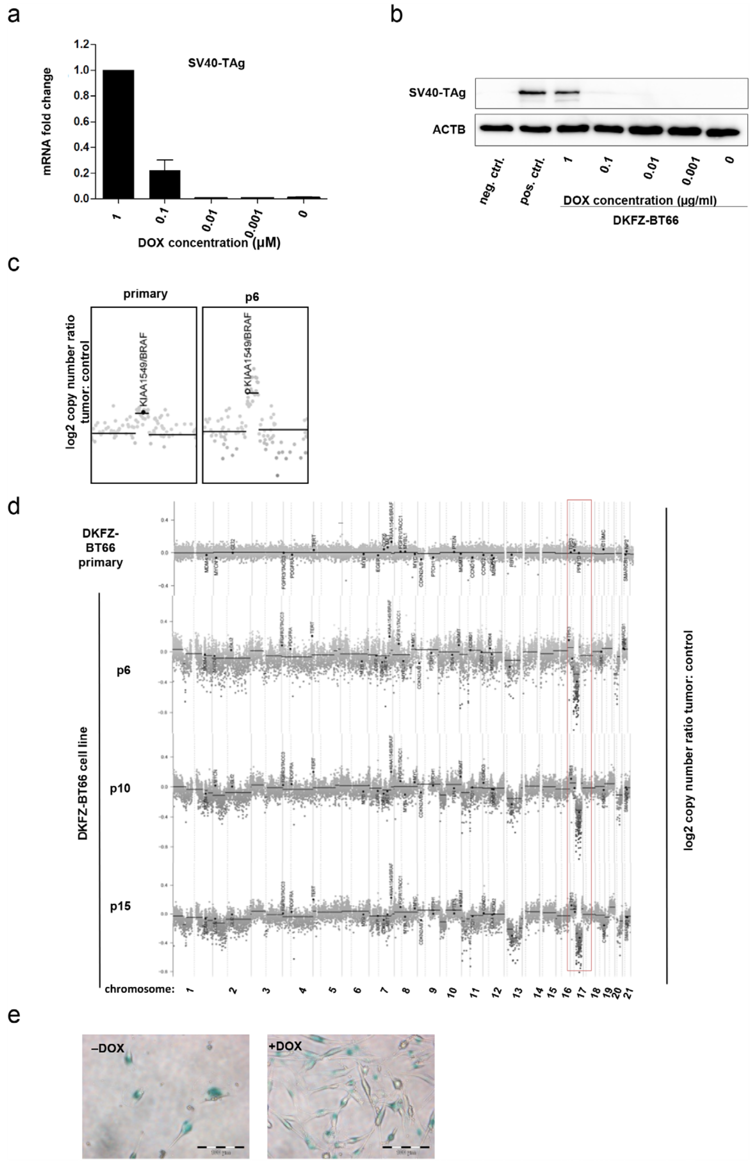

Supplementary Figure 1: a. Fold change of SV40-TAg mRNA level in DKFZ-BT66 cells after five days of culture in the indicated concentrations of doxycycline normalized to cells cultured in the presence of 1 μg/ml doxycycline. Depicted are mean +/- SD **b.** SV40-TAg protein level in DKFZ-BT66 cells cultured in the indicated concentrations of doxycycline for five days. HEK293T cells, constitutively expressing SV40-TAg, served as positive control; BT-40 glioma cells, not expressing SV40-TAg, were used as negative control. **c.** Detail of chromosome 7q from the DNA-methylation-derived copy number plot of DKFZ-BT66 primary tumor and cell line. The comparison of the height of the chromosomal gains indicates a higher content of KIAA1549:BRAF positive cells in the cell line as compared to the primary tumor. **d.** Copy number plots derived from DNA-methylation array of DKFZ-BT66 primary tumor and cultivated cells. The chromosomal aberrations (partial loss of chromosome 17 is marked as most prominent example in a red box) found in the cell line compared to the primary tumor were stable over time without gain of any new noteworthy alterations. **e.** Staining for SA-β-galactosidase in DKFZ-BT66 cells cultured in the absence of doxycycline over five days (-DOX) and in the presence of 1 μg/ml doxycycline (+DOX). SA-β-galactosidase positivity was detected in both conditions.

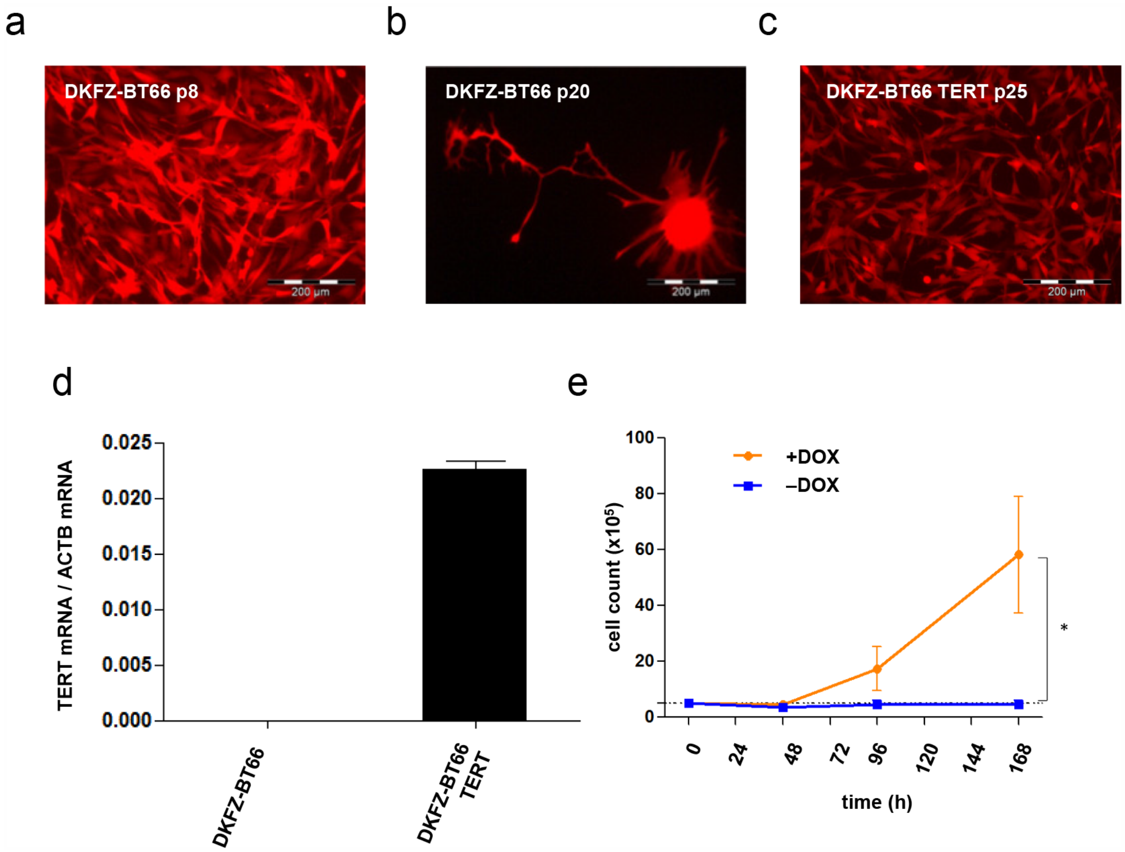

Supplementary Figure 2: a, b, c. Fluorescence microscopic pictures depicting RFP expression a) Early passage DKFZ-BT66 cells (p8= passage 8). b) Late passage DKFZ-BT66 cell (p20= passage 20) in growth arrest with enlarged and irregularly formed cell body. RFP expression is unchanged as compared to early passage cells. **c.** DKFZ-BT66 cells additionally transduced with pBabe_hygro_hTERT at passage 18 (DKFZ-BT66 TERT). The cells were able to proliferate beyond their normal life span (here p25= passage 25) and show a closer similarity to early passage cells than to arrested late passage cells not expressing TERT. **d.** Detection of TERT gene expression by RT-qPCR normalized to expression of ACTB. DKFZ-BT66 cells do not express TERT as compared to the TERT overexpressing cells. Depicted are mean +/- SD **e.** Growth kinetics of DKFZ-BT66 TERT in the presence and absence of doxycycline. DKFZ-BT66 TERT remained dependent on doxycycline for proliferation. Depicted are mean +/- SD.

Supplementary Table 1: Antibodies used for western blot analysis

antibody	company	source/clonality	dilution	catalogue number (clone)
SV40 Large T Antigen	Abcam, UK	mouse monoclonal	1:1000	ab80564 [PAb108]
MEK (1/2)	Cell Signaling Technology	rabbit monoclonal	1:1000	9122
pMEK (1/2) (Ser217/ Ser221)	Cell Signaling Technology	rabbit monoclonal	1:1000	9154
ERK (1/2)	Cell Signaling Technology	rabbit monoclonal	1:1000	4695
pERK (1/2) (Thr202/ Tyr204)	Cell Signaling Technology	rabbit monoclonal	1:1000	4377
TP53	Santa Cruz	mouse monoclonal	1:200	sc-126
CDKN1A	Santa Cruz	mouse monoclonal	1:200	sc-6246
CDKN2A	Abcam, UK	rabbit monoclonal	1:1000	108349
ACTB	Sigma- Aldrich	Mouse monoclonal	1:10.000	A5441
goat-anti-mouse IgG HRP	Dianova	polyclonal	1:10.000	115-035-003
donkey-anti-rabbit IgG HRP	Promega	polyclonal	1:10.000	V795A

Supplementary Table 2: List of RT-qPCR primers

Target	name	sequence	catalogue number	company
SV40 Large T antigen forward	SV40-TAg forward	gatgatgatgatgaagacagccagg	custom	Invitrogen
SV40 Large T antigen reverse	SV40-TAg reverse	tgatcatgaacagactgtgaggact	custom	Invitrogen
KIAA1549:BRAF-fusion (KEX16:BEX9) forward	KIAA1549 exon 16 forward	gtccttctacagcccagccca	custom	Invitrogen
KIAA1549:BRAF-fusion (KEX16:BEX9) reverse	BRAF exon 10 reverse	tggagatttctgtaaggctttcacgt	custom	Invitrogen
ACTB forward	ACTB forward	ctggaacggtgaaggtgaca	custom	Invitrogen
ACTB reverse	ACTB reverse	aagggacttcctgtaacaatgca	custom	Invitrogen
CDKN1A	Hs_CDKN1A_1_SG QuantiTect Primer Assay	/	QT00062090	Qiagen
CDKN2A	Hs_CDKN2A_1_SG QuantiTect Primer Assay	/	QT00089964	Qiagen
TERT	Hs_TERT_1_SG QuantiTect Primer Assay	/	QT00073409	Qiagen

Supplementary Table 3: The 68 most differentially regulated genes in the two states, proliferation (presence of doxycycline) and growth arrest (absence of doxycycline), were included in the PA OIS signature. Direction of gene regulation (up vs. down) is shown for cells in the arrested state.

See Supplementary File 1
